# Experimental Study of Reciprocating Friction between Rape Stalk and Bionic Nonsmooth Surface Units

**DOI:** 10.1155/2015/627960

**Published:** 2015-09-28

**Authors:** Zheng Ma, Yaoming Li, Lizhang Xu

**Affiliations:** Key Laboratory of Modern Agricultural Equipment and Technology, Ministry of Education and Jiangsu Province, Jiangsu University, Zhenjiang, Jiangsu 212013, China

## Abstract

*Background*. China is the largest producer of rape oilseed in the world; however, the mechanization level of rape harvest is relatively low, because rape materials easily adhere to the cleaning screens of combine harvesters, resulting in significant cleaning losses. Previous studies have shown that bionic nonsmooth surface cleaning screens restrain the adhesion of rape materials, but the underlying mechanisms remain unclear. *Objective*. The reciprocating friction between rape stalk and bionic nonsmooth metal surface was examined. *Methods*. The short-time Fourier transform method was used to discriminate the stable phase of friction signals and the stick-lag distance was defined to analyze the stable reciprocating friction in a phase diagram. *Results*. The reciprocating friction between rape stalk and metal surface is a typical stick-slip friction, and the bionic nonsmooth metal surfaces with concave or convex units reduced friction force with increasing reciprocating frequency. The results also showed that the stick-lag distance of convex surface increased with reciprocating frequency, which indicated that convex surface reduces friction force more efficiently. *Conclusions*. We suggest that bionic nonsmooth surface cleaning screens, especially with convex units, restrain the adhesion of rape materials more efficiently compared to the smooth surface cleaning screens.

## 1. Introduction

China is the largest producer of rape oilseed in the world with an annual production of 12 million tons, which accounts for approximately 35% of global rape oilseed production [1]. However, compared to other major producing areas or countries in the world, the mechanization level of rape harvest in China is relatively low (under 16.88%), and the rapeseed is still harvested manually, because the rape combine harvesters that are currently used in China have cleaning systems with poor performance.

**Figure 1:**
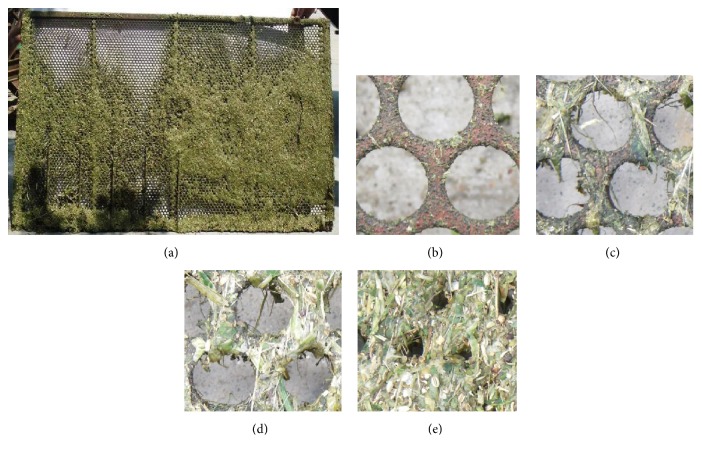
Rape threshing materials adhere to the cleaning screens of combine harvesters resulting in clogging.

The Yangtze River area is the main rapeseed-producing region of China. In this region, temperature and relative humidity levels are very high during rape harvesting, and the water content of rape stalks is about 70%–80%. Therefore, the relatively damp rape materials easily adhere to the cleaning screens of combine harvesters resulting in high cleaning losses (Figure 1) [2, 3].

**Figure 2:**
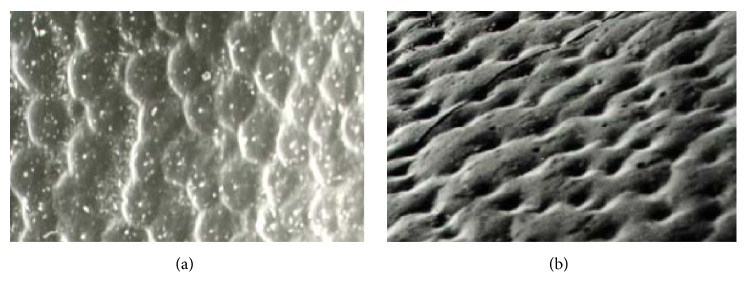
Convex surface of dung beetle's head (a) and concave surface of dung beetle's chest (b).

In recent years, bionic research continuously provides efficient methods or ideas for solving engineering problems [4, 5]. Rape material adherence to cleaning screens can be classified as an adhesion and friction problem between damp materials and moving metal parts [3]. In this field, previous studies on adhesion, friction, and bionics indicated that some soil animals with nonsmooth body surfaces, such as dung beetles (Figure 2), pass through damp and sticky materials with less adhesion [6, 7]. Tong et al. studied the abrasive wear behavior of pangolin scales to provide reference and basis for friction-reducing research between soil and soil-touching parts of agriculture machinery [8]. Other researchers used plough and bulldozing plates with nonsmooth surface units to conduct field experiments and showed that they all had nonadhesive and wear-resistance characteristics [9, 10].

In a previous study, we developed a bionic nonsmooth surface cleaning screen and compared it in the field to a common smooth surface cleaning screen [11, 12]. The performances of three rape combine harvesters of the same model equipped with three different cleaning screens were used under the same field conditions. One of the combine harvesters was equipped with a common smooth surface cleaning screen, while the other two combine harvesters had bionic nonsmooth surface cleaning screens with concave or convex units, respectively. About 70% of the common smooth surface cleaning screen area was clogged every 5 acres of continuous rape harvest resulting in high cleaning losses, while the bionic nonsmooth surface cleaning screens with concave and convex units could continuously harvest 12 and 23 acres of rape, respectively [4, 5]. The results showed that bionic nonsmooth surface cleaning screens could restrain the adhesion of rape materials, but the underlying mechanism remains unclear. In this study, we conducted a reciprocating friction experiment and the stable friction data were analyzed, in order to investigate whether the bionic nonsmooth surface cleaning screens are efficient because they reduce friction or repel adhesion or both.

## 2. Materials and Methods

Rape stalks with high water content (72% ± 4%) were used in this study as upper specimens, because they are the main threshing materials that adhere to cleaning screens in combine harvesters. A reciprocating friction form simulated the actual movement of cleaning screens, and surface units of the lower specimen were consistent with those used in the field experiment [4, 5]. All tests were repeated three times.

### 2.1. Experimental Test-Rig

**Figure 3:**
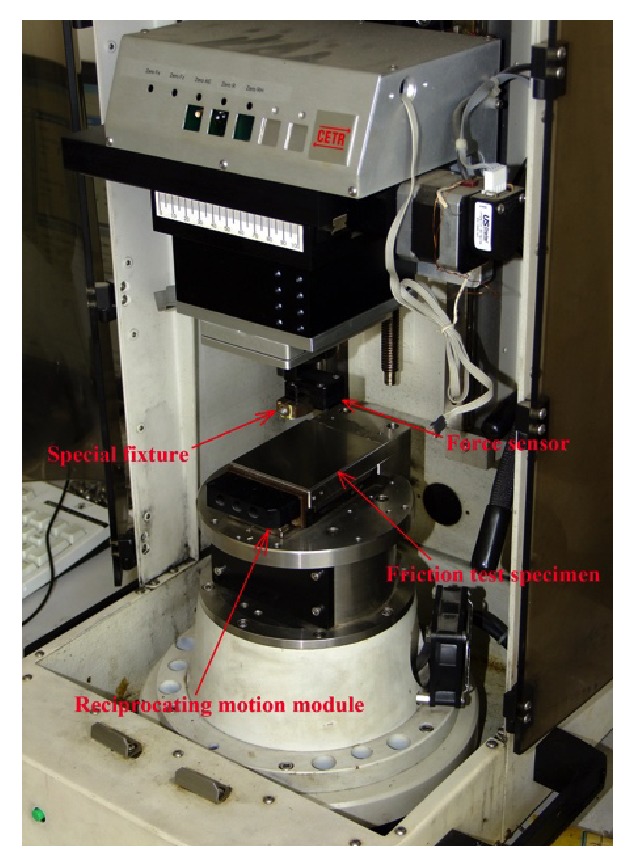
A UMT-2 type friction tester.

UMT-2 type friction tester and a high-speed-reciprocating compressor (CETR, USA) were used to study the reciprocating friction between rape stalk and bionic nonsmooth metal surface (Figure 3). The range of reciprocating frequency of the compressor was 0–50 Hz, and the unidirectional maximum stroke was 30 mm. The measuring range of the force sensor used in the tester was 0–500 g with a 0.1 g resolution, and the sampling rate was 100 Hz.

Under actual operating conditions, the unidirectional stroke range of cleaning screens is 20–40 mm, and the reciprocating frequency is 4–6 Hz. Based on preliminary tests, four kinds of crank speeds (100 rpm, 300 rpm, 500 rpm, and 700 rpm) were applied to investigate the effect of reciprocating frequency (corresponding to 1.67 Hz, 5.0 Hz, 8.33 Hz, and 11.67 Hz). Due to poor wear-resistance of rape stalk, the upper specimen was replaced with a fresh one every 10 s. The experiment was conducted in a laboratory at 22°C to minimize the effect of temperature on the properties of rape stalk.

### 2.2. Special Fixture

**Figure 4:**
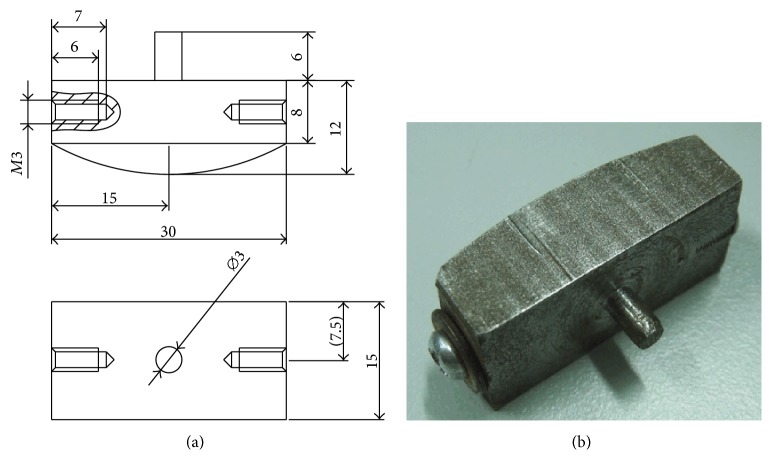
Dimensional drawing and photograph of the special fixture developed to hold the rape stalk.

In order to meet the requirements of the friction test, a special metal fixture was developed to hold the rape stalk. Two threaded holes were designed at both ends of the special fixture to fix the rape stalk, and the lower surface of the special fixture was curved to reduce the contact area between upper and lower specimens and minimize the effect of contact area during the reciprocating friction process. On the top of the special fixture, a cylinder was connected with the force sensor of the friction tester. A dimensional drawing and photograph of the special fixture are shown in Figure 4.

### 2.3. Friction Samples and Test Method

**Figure 5:**
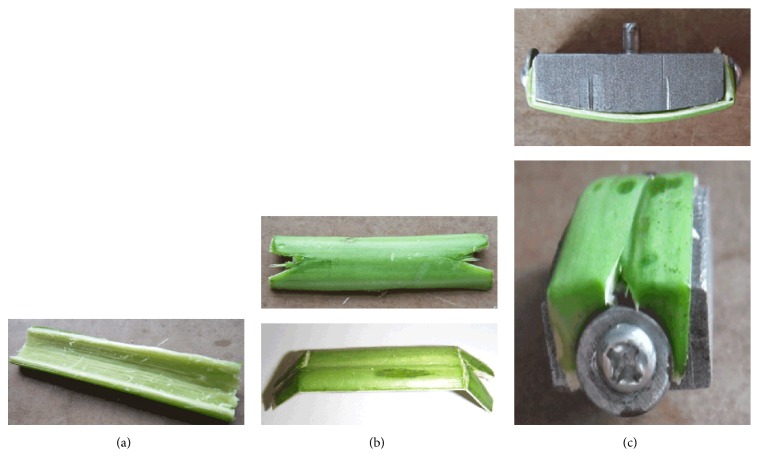
The process of manufacturing and installation of rape stalk sample (upper specimen).

In the reciprocating friction test, the upper specimen was fresh rape stalk obtained during harvest and fixed at the bottom curved surface of the special fixture. Each rape stalk was cut into a rectangular shape (length 50 mm, width 10 mm) and a V-shape cut was made at each end (Figures 5(a) and 5(b)). The rape stalk was bent toward the inside surface in order for the outside to be used as the friction surface and was fixed on the special fixture with two side screws (Figure 5(c)).

**Figure 6:**
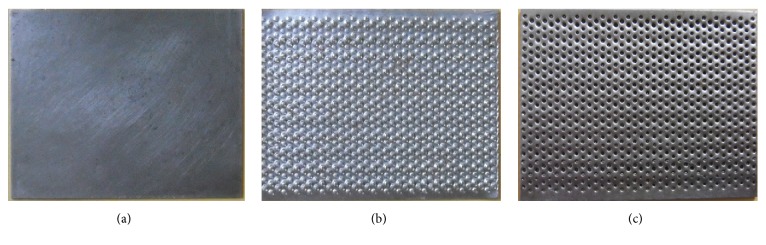
Photograph of three different rectangular plates with (a) smooth, (b) convex, and (c) concave surfaces.

**Figure 7:**
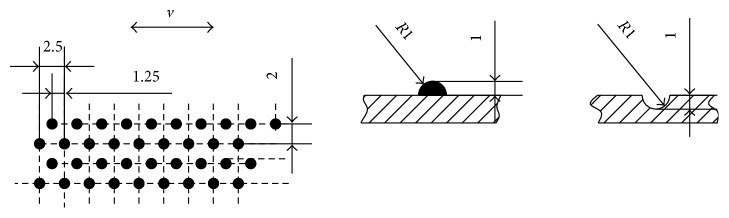
Size of the nonsmooth surface units.

The lower specimens were three rectangular metal plates (length 75 mm, width 60 mm) with different surface morphology (Figure 6), one with smooth surface (Figure 6(a)) and the other two with nonsmooth surfaces (Figures 6(b) and 6(c)). The size of the nonsmooth units is shown in Figure 7. Before the test, the metal plates underwent stainless process and surface drying process.

The friction test was conducted between the upper specimen (fresh rape stalk) and the lower specimen (one of the three types of metal plates) at four different reciprocating frequencies (1.67 Hz, 5.0 Hz, 8.33 Hz, and 11.67 Hz).

## 3. Result and Discussion

### 3.1. TFA of Reciprocating Friction

**Figure 8:**
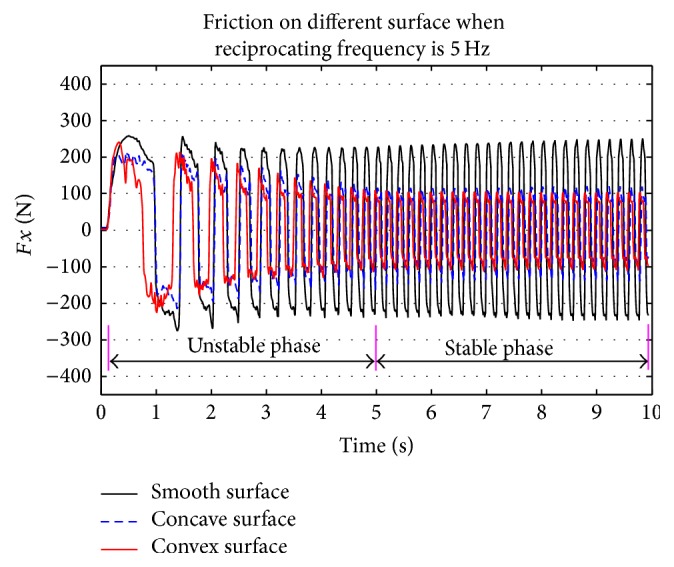
Friction force between rape stalk and three different metal plates at reciprocating frequency of 5 Hz.

Figure 8 shows parts of time-varying friction force curves obtained in this study at a reciprocating frequency of 5.0 Hz. To discriminate when the friction force curves turned into the stable phase (both in amplitude and frequency), time-frequency analysis (TFA) was performed to transform friction forces and identify the stable phase.

TFA is a basic analysis method that allows designing a two-dimensional joint distribution function *P*(*t*, *ω*) of time *t* and frequency *ω* and obtaining the energy distribution of unstable signals at a certain frequency and time range. Under ideal conditions, the time-frequency distribution function *P*(*t*, *ω*) structured by signal *s*(*t*) is described as follows:

(1) ∫−∞+∞Pt,ωdω=st2,

(2) ∫−∞+∞Pt,ωdt=12πs^ω2,

where (1) shows that the summation of the signal energy of all frequencies at a special time is the energy density (instantaneous power) of the signal at the special time and (2) shows that the summation of the signal energy of all times at a special frequency is the spectral density of the signal at the special frequency. Although there are many TFA methods such as Wigner-Ville distribution, Wavelet transform, and Hilbert-Huang transform, the classic short-time Fourier transform (STFT) was the most efficient for studying the friction signals and discriminating the stable phase [13].

STFT is a basic and widely used TFA method, which was proposed by Gabor. The basic concept of STFT is to study unstable signals as an accumulation of short-time series of stable signals in the frame of Fourier transform (FT) using the window function, which is nonzero for a short period of time [14–17]. STFT method is described as follows:

(3) $$\begin{array}{c} {\text{STFT}}_{s}\left(\;t,\omega \;\right)=\overset{+\infty }{\underset{-\infty }{\int }}s\left(\;\tau \;\right)h\left(\;\tau -t\;\right)e^{-i\omega \tau }d\tau , \end{array}$$

where *s*(*t*) is the unstable signal and *h*(*t*) is the window function. To compute discrete signals, the discrete STFT (DSTFT) method was used to transform the friction force, and hamming window was selected as a window function to avoid the boundary effect of the rectangular window. DSTFT method is described as follows:

(4) $$\begin{array}{c} \text{STFT}\left(\;n,k\;\right)=\overset{+\infty }{\underset{m=-\infty }{\sum }}s\left(\;m\;\right)h\left(\;n-m\;\right)e^{-i\left(2\pi /N\right)mk}. \end{array}$$

**Figure 9:**
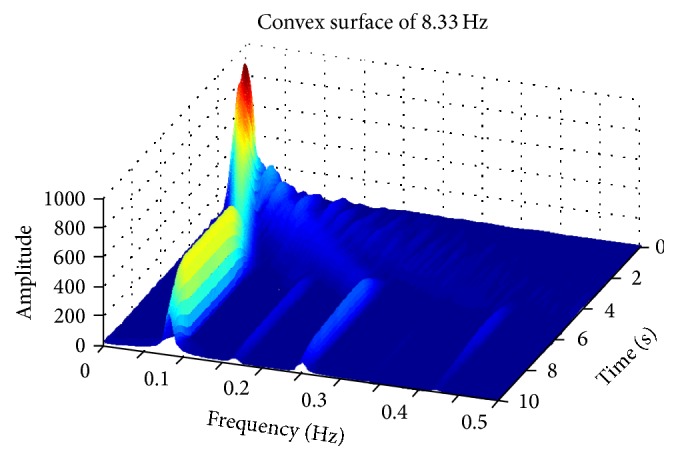
Three-dimensional time-frequency distribution of friction force between convex surface and rape specimen when reciprocating frequency is 8.33 Hz.

Figure 9 shows the three-dimensional time-frequency distribution of friction force between bionic convex metal surface and rape specimen, when reciprocating frequency was 8.33 Hz. The frequency of friction signals was increased significantly and amplitude of the friction signals decreased quickly between 0 and 5 s. After 5 s, the main frequency and amplitude of friction signals became stable. When all the friction forces of the test were transformed using the STFT method (as shown in the appendix), it was revealed that after 5 s all of the friction forces in the test transformed into the stable phase, and these signals were used for additional analysis.

### 3.2. Analysis of Stable Friction Force

**Figure 10:**
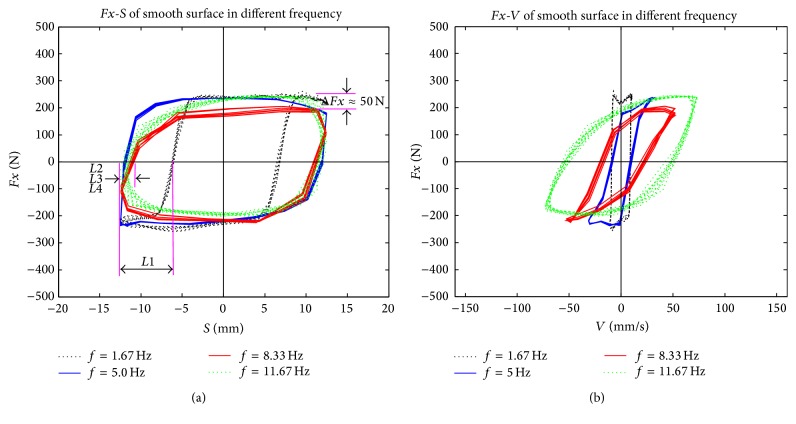
The patterns of (a) relative displacement and (b) relative velocity during the stable friction process of smooth surface at four different reciprocating frequencies.

**Figure 11:**
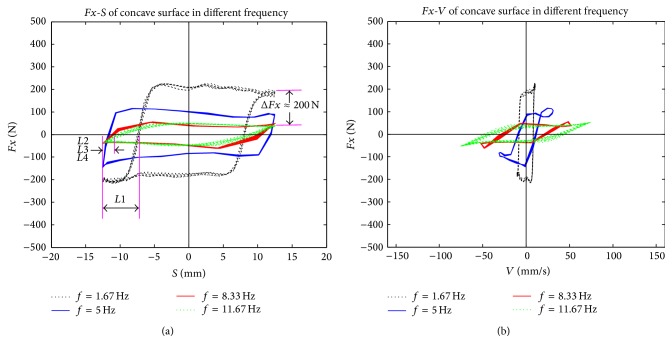
The patterns of (a) relative displacement and (b) relative velocity during the stable friction process of concave surface at four different reciprocating frequencies.

**Figure 12:**
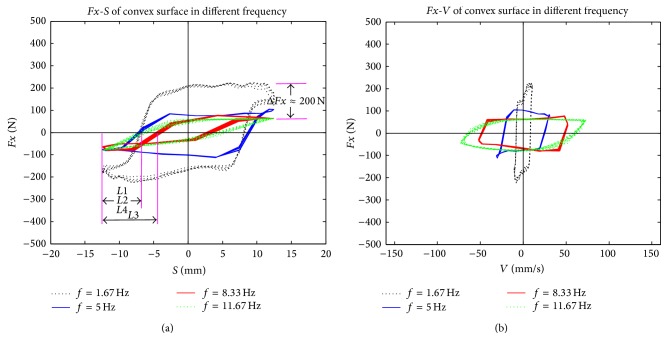
The patterns of (a) relative displacement and (b) relative velocity during the stable friction process of convex surface at four different reciprocating frequencies.

To study the relationship between friction force and relative movement parameters during the reciprocating friction process, relative displacement and relative velocity were deduced. The reciprocating movement is a sinusoidal motion, which is described as follows:

(5) S=r·cosωt,V=−ωr·sinωt,

where *S* is relative displacement, *V* is relative velocity, *r* is amplitude (in this study *r* = 12.5 mm), and *ω* is circular frequency (in this study *ω* = 10.48, 31.4, 52.31, or 73.29). The patterns of relative displacement and relative velocity during the stable friction process of four different reciprocating frequencies were examined (Figures 10–12).

**Figure 13:**
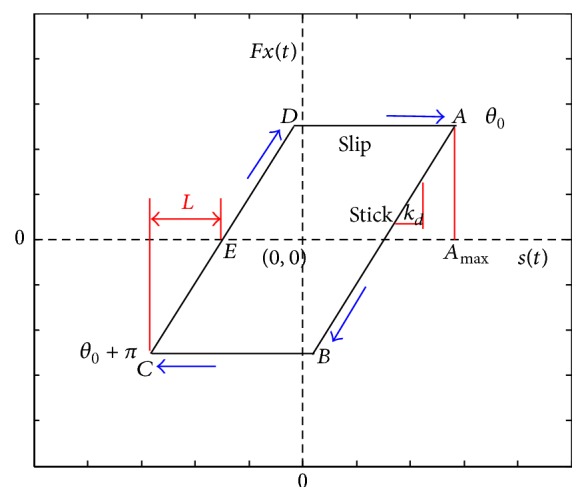
Phase diagram of the ideal stick-slip friction.

The patterns of relative displacement between rape stalk and three different metal surfaces were typical parallelogram closed curves, which indicated that reciprocating friction between rape stalk and metal surface included a typical stick friction. Wang and Chen and Al Sayed et al. reported that there are two friction phases in an ideal stick-slip friction (slip-friction and stick-friction), which are described as follows (Figure 13) [18, 19]:

(6) Fxt=±μ·Fnslipphase±μ·Fn+kdst−Amaxstickphase,

where *k*_*d*_ is the slope of segment *AB*, *F*_*n*_ is the normal force vertical to friction force, *A*_max_ is the maxim amplitude of reciprocating displacement, and *s*(*t*) is the function of *θ*_0_. The relationship between these parameters is described as follows:

(7) $$\begin{array}{c} A_{max}=s\left(\;\theta_{0}\;\right)=\overset{N}{\underset{n=1}{\sum }}\left[\;a_{n}\cos\left(\;n\theta_{0}\;\right)+b_{n}\sin\left(\;n\theta_{0}\;\right)\;\right], \end{array}$$

where angle signal is used to estimate whether the friction is in slide-friction phase or in stick-friction phase, *θ*_0_ is the turning angle when vector $\vec{DA}$ turns to $\vec{AB}$, and *θ*_0_ + *π* is the whole turning angle when $\vec{DA}$ turns to $\vec{CD}$ in a clockwise direction (as the arrows show in Figure 13). The vectors of $\vec{DA}$ and $\vec{BC}$ are the forward and backward slip-friction phases, respectively, and vectors of $\vec{AB}$ and $\vec{CD}$ are the forward and backward stick-friction phases, respectively.

Although the phase diagrams of friction forces shown in Figures 10–12 and the phase diagram of the ideal stick-slip friction shown in Figure 13 seem the same, there are some differences between them, because of test errors. Some nonlinear mechanisms that exist in reciprocating frictions result in strong nonlinear relationships between friction forces and other parameters, such as normal forces, amplitude, frequencies, and surface features [20]. Although it is possible that an unknown mechanism exists between biological materials (rape stalk) and metal surfaces with bionic nonsmooth units, we suggest that the reciprocating friction between rape stalk and metal surface is a typical stick-slip friction.

### 3.3. Effect of Reciprocating Frequency

As shown in Figure 9, amplitude of friction force between rape stalk and smooth metal surface showed almost no fluctuation with the rise of reciprocating frequency (Δ*Fx* ≤ 50 N). However, the amplitude of friction force between rape stalk and bionic nonsmooth metal surface (Figures 11 and 12) decreased significantly with the increase of reciprocating frequency (Δ*Fx* ≈ 200 N). These results indicate that bionic nonsmooth surface cleaning screens may reduce friction more efficiently compared to the common smooth surface cleaning screens.

In this study, the horizontal distance between *C* and *E* (as shown in Figure 13) was defined as the stick-lag distance *L* that characterized the stick level of the stick-slip friction force, which showed that the direction of displacement and the direction of friction force did not change at the same time. Stick-lag distance *L* was used to analyze stable reciprocating friction (Figures 10–12), where *L*1, *L*2, *L*3, and *L*4 were the stick-lag distances at 1.67 Hz, 5.0 Hz, 8.33 Hz, and 11.67 Hz, respectively. The stick-lag distances of smooth metal surface and of concave metal surface at low frequency (1.67 Hz) are longer than those at high frequency (5.0 Hz, 8.33 Hz, and 11.67 Hz) and *L*1 > *L*2 ≈ *L*3 ≈ *L*4 (Figures 9 and 10). However, the stick-lag distance of convex metal surface at a frequency of 8.33 Hz is longer than those at 1.67 Hz, 5.0 Hz, and 11.67 Hz and *L*1 ≈ *L*2 < *L*3 > *L*4 (Figure 11). An increase in reciprocating frequency leads to a decrease in stick-lag distance of smooth metal surface and of concave metal surface and an increase in the stick-lag distance of convex metal surface.

Overall, compared to smooth metal surface, the concave metal surface and convex metal surface may decrease the friction force with the increase in reciprocating frequency, but only the convex metal surface can increase the stick-lag distance with the reciprocating frequency. Overall, our results suggest that bionic nonsmooth surface cleaning screens, especially with convex units, restrain the adhesion of rape materials more efficiently compared to the smooth surface cleaning screens.

## 4. Conclusions

In this study, reciprocating friction test and data analysis were performed to examine why cleaning screens with bionic nonsmooth surfaces (especially with convex units) restrain the adhesion of damp rape materials more efficiently compared to the smooth surface cleaning screens. Overall, the results of this study showed the following:

(i) The reciprocating friction between the rape stalk and metal surface is a typical stick-slip friction, but the underlying nonlinear mechanism remains to be studied.
(ii) The friction force between the bionic nonsmooth metal surface with concave or convex units and rape stalk decreased about 200 N with the increase in reciprocating frequency, but the friction force between the smooth metal surface and rape stalk did not fluctuate with the increase in reciprocating frequency.
(iii) Changes in stick-lag distance *L* of concave metal surface to rape stalk are similar to those of smooth metal surface, but the stick-lag distance of convex metal surface to rape stalk increases with the reciprocating frequency. These results may explain the reason that the cleaning screens with convex surface units are more efficient than those with concave surface units.

## Figures and Tables

**Figure 14:**
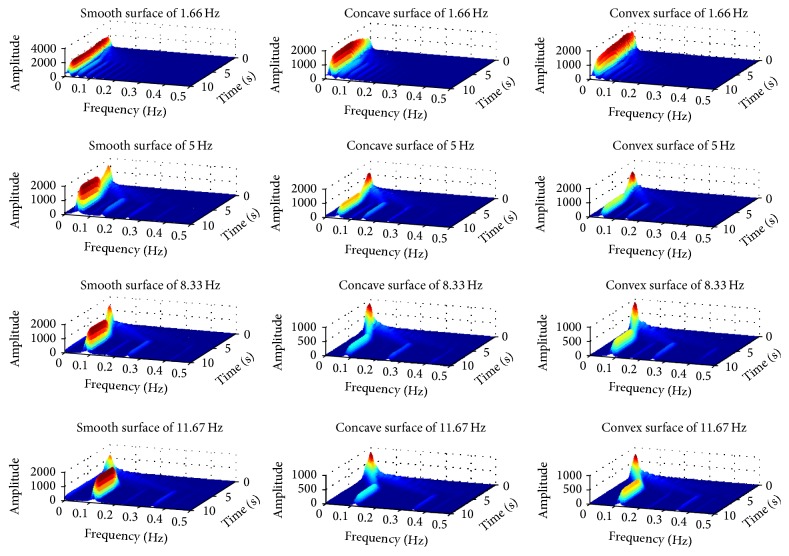


## Appendix

See Figure 14.
